# Identification and validation of the inflammatory response-related LncRNAs as diagnostic biomarkers for acute ischemic stroke

**DOI:** 10.1038/s41598-025-98101-0

**Published:** 2025-04-22

**Authors:** Peidong He, Hongxiang Jiang, Jiangrui Zhu, Min Hu, Ping Song

**Affiliations:** 1 Renmin Hospital of Wuhan University, Wuhan, 430060 China; 2 Department of Neurosurgery, Renmin Hospital of Wuhan University, 238 Jiefang Road, Wuchang Distict, Wuhan, 430060 Hubei Province China; 3 Department of Gynecology and Obstetrics, Renmin Hospital of Wuhan University, Wuhan, 430060 China

**Keywords:** MALAT1, GAS5, Diagnostic biomarkers, Inflammatory response, Acute ischemic stroke, Computational biology and bioinformatics, Neuroscience

## Abstract

Ischemic stroke is one of the leading causes of deaths and disability, which is linked to inflammation. In this study, we aimed to identify inflammation-related lncRNAs as diagnostic biomarkers of acute ischemic stroke (AIS). A competing endogenous RNAs (ceRNA) network was established through whole transcriptome analysis. Gene expression datasets from the GEO database were analyzed to identify differentially expressed genes (DEGs), miRNAs and lncRNAs. Inflammation-related DEGs were determined through the intersection of the DEGs of the inflammation-related gene set from Genecards. Multiple databases like lncBase and Targetscan were analyzed to establish a ceRNA network. Several hub genes and sub-networks were obtained from a protein to protein (PPI) network. In addition, the candidate lncRNAs derived from the subnetwork were validated using mice MCAO model and clinical samples. Finally, a network comprising 20 lncRNAs, 26 miRNAs, and 43 inflammatory genes was analyzed, leading to the identification of MALAT1, SNHG8, and GAS5 as potential diagnostic biomarkers. Knockdown of MALAT1 and GAS5 resulted in decreased neurological severity score and inflammation response in mice MCAO model, indicating that these genes were significant diagnostic biomarkers for distinguishing AIS from healthy controls. These findings show that circulating MALAT1 and GAS5 have the potential to serve as clinical diagnostic biomarkers of AIS associated with inflammation.

## Introduction

As the global aging population increases, the prevalence of risk factors for cerebrovascular disease and stroke has been on the rise globally, causing significant deaths and disability burden worldwide^1^. Ischemic stroke (IS) is estimated to affect 87% of all types of stroke and is the second major cause of death and the third-leading cause of disability^2,3^. Currently, IS is treated through the administration of recombinant tissue-type plasminogen activator to restore vascular patency and the blood flow by lysing blood clots. However, thrombolytic therapy is only effective within a narrow timeframe of 3 to 4.5 h following the onset of symptoms^4^. Moreover, when applied outside this timeframe, it not only diminishes its efficacy but also increases the risk of secondary hemorrhage^5^. The adopted diagnostic approach for acute ischemic stroke (AIS) involves a combination of clinical symptoms and neuroimaging modalities, including computed tomography (CT) and magnetic resonance imaging (MRI). However, the application of MRI requires a long imaging time, which can potentially miss the optimal timing for intravenous administration or mechanical thrombolysis. Furthermore, CT is less sensitive following AIS and may not detect early changes associated with the disease^6^. Identifying novel genetic characteristics or biomarkers could offer valuable insights for the timely treatment of AIS. Meanwhile, prompt and accurate diagnosis of patients with AIS may promote the treatment of patients and improve disease prognosis.

Competing endogenous RNAs (ceRNA) have been implicated in diverse biological mechanisms. In summary, ceRNAs function as sponges, competing or synergizing with microRNA (miRNA) through miRNA response elements (MREs), to modulate the expression of miRNA target genes, functioning as post-transcriptional regulators of gene expression^7^. In recent years, new ceRNA regulatory network have been proposed based on miRNA expression to investigate the regulation of miRNA-targeted genes. Noncoding RNAs (ncRNAs) are transcripts that are not translated into proteins, which include miRNA and long noncoding RNA (lncRNA)^8^. They have been implicated in diverse biological processes, including cell cycle regulation and immunological response, making it an important contributor to the development of cancer, neurological illnesses, and cardiovascular diseases^9^. LncRNAs are ncRNAs with more than 200 nucleotides and is involved in the regulation of gene expression by directly interacting with mRNAs as transcriptional regulators or functioning as ceRNAs^2,10^. Several lncRNAs involved in neuroinflammation have been documented in AIS^11^. This indicates that lncRNAs can help to differentiate AIS patients from health controls (HCs).

Neuroinflammation is caused by complex innate immune responses activated by reactive oxygen species, and is known to disrupt homeostasis. Secondary messengers, reactive oxygen species, chemokines, and proinflammatory cytokines have been shown to trigger inflammation^12^. Research has demonstrated that microglias and astrocytes are activated shortly after IS, releasing large amounts of cytokines and chemokines^13^ and infiltrating leukocytes^14,15^. Neuroinflammation can participate in the pathogenesis of AIS and influence the prognosis of patients, potentially disrupting the blood-brain barrier, inducing brain injuries, neuronal damage, and vascular aging^7^. Notably, the development of inflammatory response following AIS occurs within hours, days, or weeks. Therefore, the application of targeted treatments and interventions to prevent post-stroke inflammation should be conducted within a specific timeframe. This implies that prompt interventions to mitigate inflammatory response following AIS are urgently needed^16^. Numerous studies have explored various inflammatory biomarkers for diagnosing ischemic cerebrovascular disease^17^. However, few diagnostic biomarkers have been proven to serve as potential diagnostic markers of AIS. Therefore, it is imperative to identify new immunoinflammatory biomarkers for predicting AIS.

In this study, we collected gene, lncRNA, and miRNA expression profiles from AIS and HCs samples from the Gene Expression Omnibus (GEO) database. The obtained data were used to construct a ceRNA network of AIS. The key lncRNAs, miRNAs, and genes in AIS were identified. The receiver operating characteristic curves (ROCs) were constructed to test the diagnostic performance of the network. Finally, two lncRNAs were found to be candidate diagnostic biomarkers, which were validated using mice middle cerebral artery occlusion (MCAO) models.

## Methods and materials

### Data collection

The datasets of AIS were obtained from the GEO database (https://www.ncbi.nlm.nih.gov/geo/), a publicly accessible collection of functional genomics data. The inclusion criteria for the dataset were as follows: the species was homo sapiens, the samples contained at least two groups, AIS patients and HCs, with a minimum of 3 samples in each group, the samples were derived from peripheral blood samples within 24 h. In the final analysis, 6 datasets comprising 3 lncRNA expression profiles (GSE122709, GSE140275, and GSE198710), 2 miRNA expression profiles (GSE110993 and GSE231431), and 1 gene expression profile (GSE16561) were obtained. A lncRNA dataset (GSE102541) was included as the validation set. The detailed information is presented in (Table 1).

**Table 1:** Characteristics of the expresion profiles.

Dataset	Platform	Type	Sample Size		Author	Year
			Stroke	Control		
GSE122709	GPL20795	lncRNA	5	5	Yan et al	2019
GSE140275	GPL16791	lncRNA	3	3	Li et al	2019
GSE198710	GPL21827	lncRNA	5	5	Jiang et al	2022
GSE110993	GPL15456	miRNA	20	20	Northoff et al	2018
GSE231431	GPL16791	miRNA	21	14	Azar et al	2023
GSE16561	GPL6883	Gene	39	24	Barr et al	2010

### Analyses of differential expression

To identify differentially expressed lncRNAs (DELs), differentially expressed miRNAs (DEMIs), and differentially expressed genes (DEGs), differential analyses were conducted on lncRNA, miRNA, and gene expression profiles derived from peripheral blood samples of individuals with AIS and HCs using “limma” in R package (v3.52.0). Next, different statistical criteria were adopted to explore gene expression profiles and variation signatures of ncRNA in each dataset. Specifically, *p* < 0.05, |log twofold change (FC)|> 0.5 was selected as the threshold for the identification of DEMIs and DEGs, and *p* < 0.05, |log twofold change|> 1 was selected as the accepted threshold for the identification of DELs. To identify differentially expressed lncRNAs (DELs) and miRNAs (DEMIs) across multiple datasets, we employed the “RobustRankAggreg” R package. For DELs, we applied stringent criteria (*p* < 0.05 and |log2-fold change|> 1), while for DEMIs, we used slightly relaxed thresholds (*p* < 0.05 and |log2-fold change|> 0.5). This approach helped minimize batch effects and other non-essential variations across the datasets, ensuring robust and reliable results. . Subsequently, the R packages “ggplot2” (v3.3.6) and “pheatmap” (v1.0.12) were utilized to plot volcano plots and heatmaps of DELs, DEMIs, and DEGs.

### Inflammatory response-related genes (IRRGs)

Inflammatory response-related genes were extracted from the Genecards database (https://www.genecards.org/). Finally, 2925 IRRGs were integrated into the gene set and by intersecting DEGs with IRRGs, inflammatory response-related DEGs (IRRDEGs) were identified.

### Construction of inflammatory response-related ceRNA network (IRRCN) of AIS

An inflammatory response-related ceRNA network of AIS mediated by lncRNAs was constructed using multiple databases to identify the target lncRNA and target genes of DEMIs. Initially, the lncRNA database lncBase^18^ was screened to search for the target lncRNAs of DEMIs. Subsequently, the identified target lncRNAs were intersected with DELs to obtain DEMI-DEL pairs. This was followed by analysis of the Targetscan^19^ and miRDB^20^ databases to identify target genes for DEMIs, which were overlapped with DEGs to generate DEMI-DEG pairs. Using the obtained DEMI-DEL and DEMI-DEG pairs, we constructed the DEL-DEMI-DEG pairs, in which DEGs and DELs share the same DEMIs. Finally, the DEL-DEMI-DEG pairs were visualized with Cytoscape (v3.10.0).

### Functional and pathway enrichment analyses

Gene ontology (GO) analysis and Kyoto Encyclopedia of Genes and Genomes (KEGG) pathway analyses were performed using the “clusterProfiler” R package (v4.2.0) to predict the biological function and pathways enriched for the genes in the IRRCN. The threshold of significance for the enrichment analyses was *p* < 0.05. The enrichment analysis results were visualized using the “ggplot2” (v3.3.6) R package.

### Analysis of the protein-protein interactions (PPI) network

The genes in IRRCN were uploaded to the STRING (v12.0, http:/www.string-db.org/)^21^ database and analyzed to predict the PPI encoded by these genes as potential drivers of AIS development. A medium confidence level of 0.4 was selected as the significant criterion. The results of the STRING analysis were imported into “Cytoscape” (v3.10.0)^22^ for visualization. Using the “Cytohubba” (v0.01)^23^ tool, we calculated the MCC, Degree, Betweenness, Closeness, EPC, and Stress scores for each node in the network. The top 8 genes with the highest scores were then identified and overlapped. The intersection of these genes was considered to represent the inflammatory response-related hub genes (IRRHGs) of the PPI network.

### Identification of key biomarkers

To identify lncRNAs associated with inflammatory responses, the lncRNA-miRNA-IRRHG subnetwork was obtained from the ceRNA network and analyzed.

### Diagnostic performance of key biomarkers

The diagnostic performance of the candidate biomarkers in the discovery set and the validation set was determined using the “pROC” R package. The area under the curve (AUC) and 95% confidence intervals (CI) were calculated to verify the reliability of the diagnostic curve.

### Middle cerebral artery occlusion-reperfusion (MCAO/R) model

S-week-old C57BL/6 J male mice were purchased from Shulaibao Biotechnology Co., Ltd. The mice were kept in a house adjusted to a temperature of 22 ± 1 °C, a relative humidity of 50 ± 1%, and a 12 h light–dark cycle. All animal experiments were performed in accordance with the institutional animal care policies and procedures of Wuhan University, as well as the recommendations of the US National Institutes of Health and the ARRIVE guidelines (https://arriveguidelines.org).

The mice MCAO models were established as described previously^24^. The mice were occluded for 1 h, followed by extraction of the monofilament and irreversible ligation of the external carotid artery. In the sham group, once the mice were administered with a simple monofilament to obstruct the middle cerebral artery, the monofilament was withdrawn, and blood flow was quickly restored. Next, penumbra brain tissue was collected 24 h after reperfusion to subsequent tests. The Institutional Animal Ethics Care and Use Committee of Wuhan University Renmin Hospital approved all animal procedures (WDRM2022-KS002).

### 52,3,5-triphenyltetrazolium chloride (TTC) staining

Ischemia-reperfusion procedure was performed for 24 h followed by deep anesthetization and intracardial phosphate buffered saline perfusion. The brains were extracted using in a hurry, snap frozen for 20 min at −20 °C, and then sliced into brain slices with a thickness of 2 mm. FolThe tissues were soaked in a 2% TTC (Jiancheng Biotech) solution and then incubated for 30 min at 37 °C in a darkness.

### RNA extraction and quantitative real-time PCR (qRT-PCR)

Total RNA was isolated from mice cerebral ischemia penumbra tissue or sham-operated tissue using the TRIzol reagent (Invitrogen, Carlsbad, CA, USA). DNaseI was added to the lysis solution to prevent DNA contamination. Next, 1 ug of total RNA was taken from each sample and reverse transcribed. It was then amplified using standard PCR kits (Invitrogen, Missouri, USA). In this PCR, β-actin served as the internal gene. The primers used in this study are listed in (Table S1).

### Silencing of growth arrest specific 5 (GAS5) and metastasis associated lung adenocarcinoma transcript 1 (MALAT1) in vivo

Briefly, mice were placed into the stereotactic apparatus following anesthetization with isoflurane (3% for induction and 1.5% for maintenance during the surgical procedure under 30% oxygen and 70% nitrogen). A small hole was made at a specific location, 2 mm from the anterior iliac crest and 1.5 mm from the sagittal suture, using a microsyringe. The mice then received injections into the right lateral ventricle at a depth of 2.5 mm below the skull surface. At 48 h before MCAO exposure, lentiviruses sh-MALAT1 or its control and sh-GAS5 or its control were injected. The cationic lipid polybrene (4 ug/ul, GenePharma) was mixed with lentivirus sh-MALAT1 or its control, lentivirus sh-GAS5 or its control (10^9^ TU/mL; GenePharma, Shanghai, China), and incubated at 37 °C for 15 min prior to MCAO. The mixture (7 ul) was injected into the lateral ventricle for more than 20 min (n = 5), using microliter syringes (Hamilton CO., Reno, NV, USA).

### Nissl’s staining

The slices were dehydrated in by exposure to graded concentrations of ethanol and then stained with 0.1% toluidine blue solution for 20 min at 37 °C. Nerve cells were examined under a light microscope. Five random fields were chosen in each slice.

### Neurological function evaluation

Following successful establishment of the MCAO model, the modified neurological severity score (mNSS) was determined to assess the neurological damage in the aforementioned groups (n = 5 in each group). On a scale of 0 to 18, normal neurological function is scored at 0, and the maximum loss score is 18 points. A score of 1 denotes incapacity to complete the test or a lack of test reflexes.

### Enzyme-linked immunosorbent assay (ELISA)

The mice were administered with phosphate-buffered saline (PBS, pH 7.4, 4 °C) for transcardial perfusion after MCAO for 24 h. The infarcted area was immediately extracted, homogenized in 200 mg/mL of 0.9% normal saline, and centrifuged for 20 min at 12,000 rpm. The supernatant was collected and kept at -80 °C. The expression of tumor necrosis factor-alpha (TNF-α), interleukin 1 beta (IL-1β), interleukin 4 (IL-4), and interleukin 10 (IL-10) in brain tissue lysates was detected using commercial ELISA kits (#88-7105-22, Invitrogen, Thermo Scientific, USA) following the manufacturer’s instructions. A standard curve was constructed based on the absorbance values to calculate the final concentration of cytokines.

### Study design and participants

All participants who visited the Department of Neurology at Renmin Hospital, Wuhan University from September 2023 to February 2024, were randomly enrolled in this study. The inclusion criteria were adopted: The first symptoms appeared within 24 h; AIS was diagnosed as an acute focal neurological deficit and was made after more than 18 h, with CT imaging confirmed the diagnosis. The exclusion criteria were: patients with a history of previous stroke, severe heart failure, acute myocardial infarction, acute and chronic kidney/liver disease, peripheral vascular disease, cancer, and inflammatory or infectious diseases. We also recruited volunteers at our physical examination facility who had no history of stroke or other specific problems as health controls (HCs). Following admission, baseline data for diagnostic work, clinical aspects, medical history, and demographic details were collected. Each patient received acute treatment following the relevant clinical guidelines, which included anticoagulation, antiplatelet therapy, intravascular interventions, and intravenous thrombolytic therapy. Ultimately, our study comprised two sample groups: a validation cohort consisting of 40 AIS patients and 40 HCs. During enrollment, the medical histories of AIS patients and clinical baseline data were matched with those of HCs from verified samples. The study followed the guidelines of the Declaration of Helsinki. Informed consent was obtained from all subjects and their legal guardian. The study protocol was also approved by the Ethics Committee of Wuhan University Renmin Hospital (Approval Number: WDRM20220301C).

### RNA extraction and qRT-PCR

Before receiving any treatment, all blood samples were collected immediately after patient admission. Whole blood was collected into EDTA-K 2 plasma tubes and centrifuged for 10 min at 4 °C at 2000 × g. Next, the plasma was stored at −80 °C in separate Eppendorf tubes without RNAase until RNA extraction. Subsequently, total RNA was extracted from plasma and reverse transcribed into complementary DNA using the HiScript ® Q RT SuperMix for qPCR Kit (Vazyme, China) following the manufacturer’s instructions. A two-step qRT-PCR was performed using SYBR Premix Ex Taq TM II on the CFX96 PCR equipment (Bio-Rad, USA). The primer sequences used in this study are shown in (Table S2). The expression of target mRNA was calculated using CT technique. GAPDH served as the internal control.

### Statistical analysis

The data were analyzed using the GraphPad Prism 8.0 software. For continuous variables, the data were compared using the student’s t-test or the Mann–Whitney U-test. For multiple comparisons of continuous variables, the One-way or Kruskal–Wallis ANOVA were adopted. Pearson’s chi-squared test or Fisher’s exact test was utilized to analyze categorical variables. To identify putative lncRNAs for AIS, logistic regression analysis and multivariate logistic regression were performed. ROC analysis was employed to examine the diagnostic values of each lncRNA for AIS. A *p*-value < 0.05 was considered statistically significant.

## Results

### Identification of differentially expressed lncRNA, miRNA, gene and IRRDEGs

**Fig. 1:**
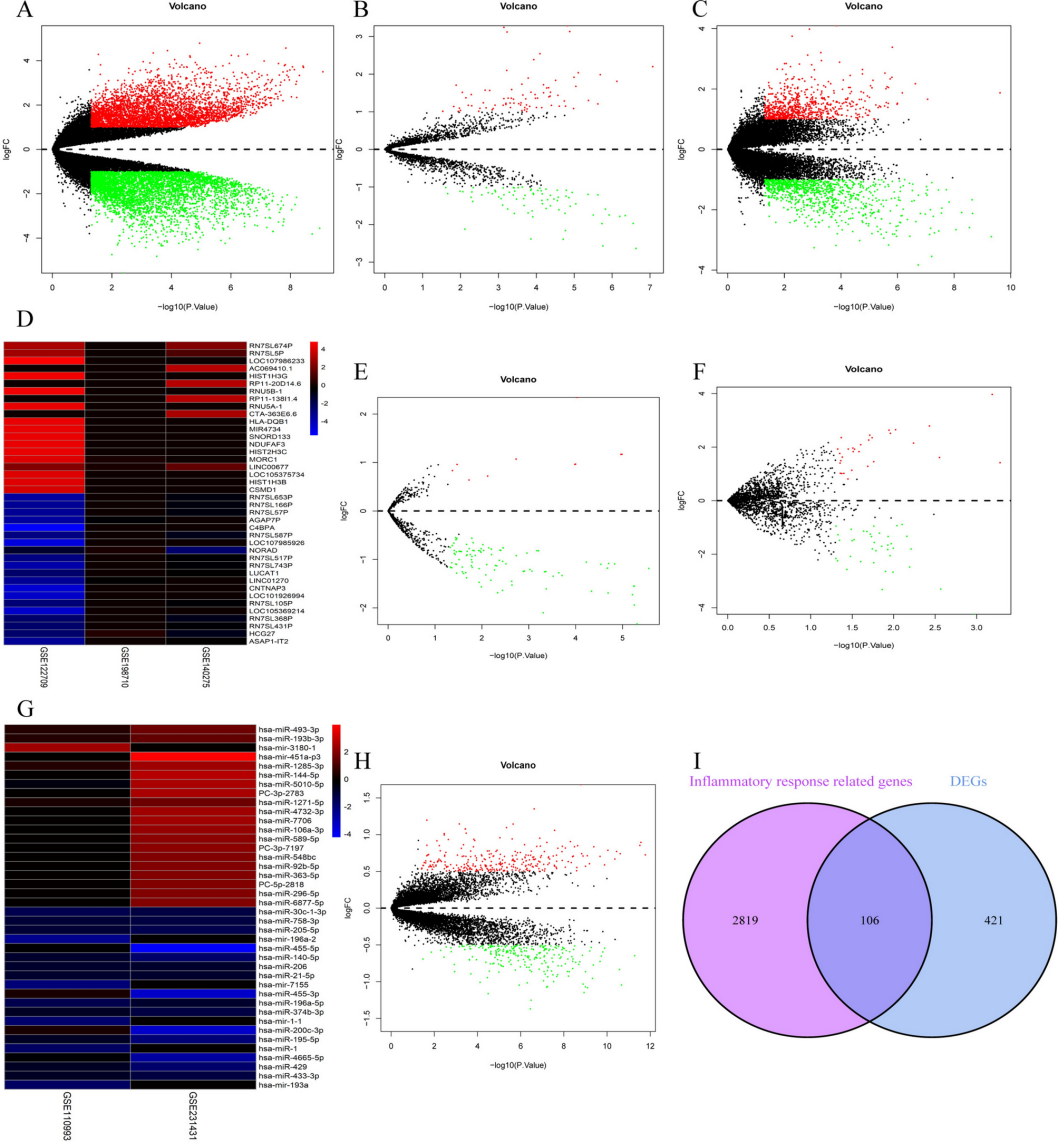
Volcano plots for differential expression analysis and heatmaps for RRA analysis. Up-regulated lncRNAs, miRNAs, and genes are represented by red plots, while down-regulated lncRNAs, miRNAs, and genes are represented by green plots. (**A**) volcano plot of GSE112709; (**B**) volcano plot of GSE140275; (**C**) volcano plot of GSE198710; (**D**) Top 20 up-regulated and down-regulated DELs for the 3 datasets identified through RRA analyse, red represents high expression and blue represents low expression. (**E**) volcano plot of GSE110993; (**F**) volcano plot of GSE231431; (**G**) Top 20 up-regulated and down-regulated DEMIs for the 2 datasets identified through RRA analyse, red represents high expression and blue represents low expression. (**H**) Volcano plot of GSE16561. (**I**) Intersection of DEGs and IRRGs.

The GSE110993 dataset containing differential expression data was downloaded from the GEO database. Moreover, we obtained the expression matrices of the other five datasets through further background correction and normalization processing. The differential expression between AIS patients and HCs was determined using the “limma” package and the differentially expressed components from different datasets were integrated. Finally, 437 DELs were identified from GSE112709, GSE140275, and GSE198710 datasets, including 270 down-regulated lncRNAs and 167 up-regulated lncRNAs (Fig. 1A–D). Further analysis obtained 215 DEMIs, which comprised 140 down-regulated miRNAs and 75 up-regulated miRNAs from the GSE11099 3 and GSE231431 datasets (Fig. 1E–G). Subsequently, 527 DEGs, including 245 down-regulated genes and 282 up-regulated genes, were identified in the GSE16561 dataset (Fig. 1H). A further analysis of the intersection between DEGs and IRRGs obtained 106 IRRDEGs (Fig. 1I).

### Construction of ceRNA network

**Fig. 2:**
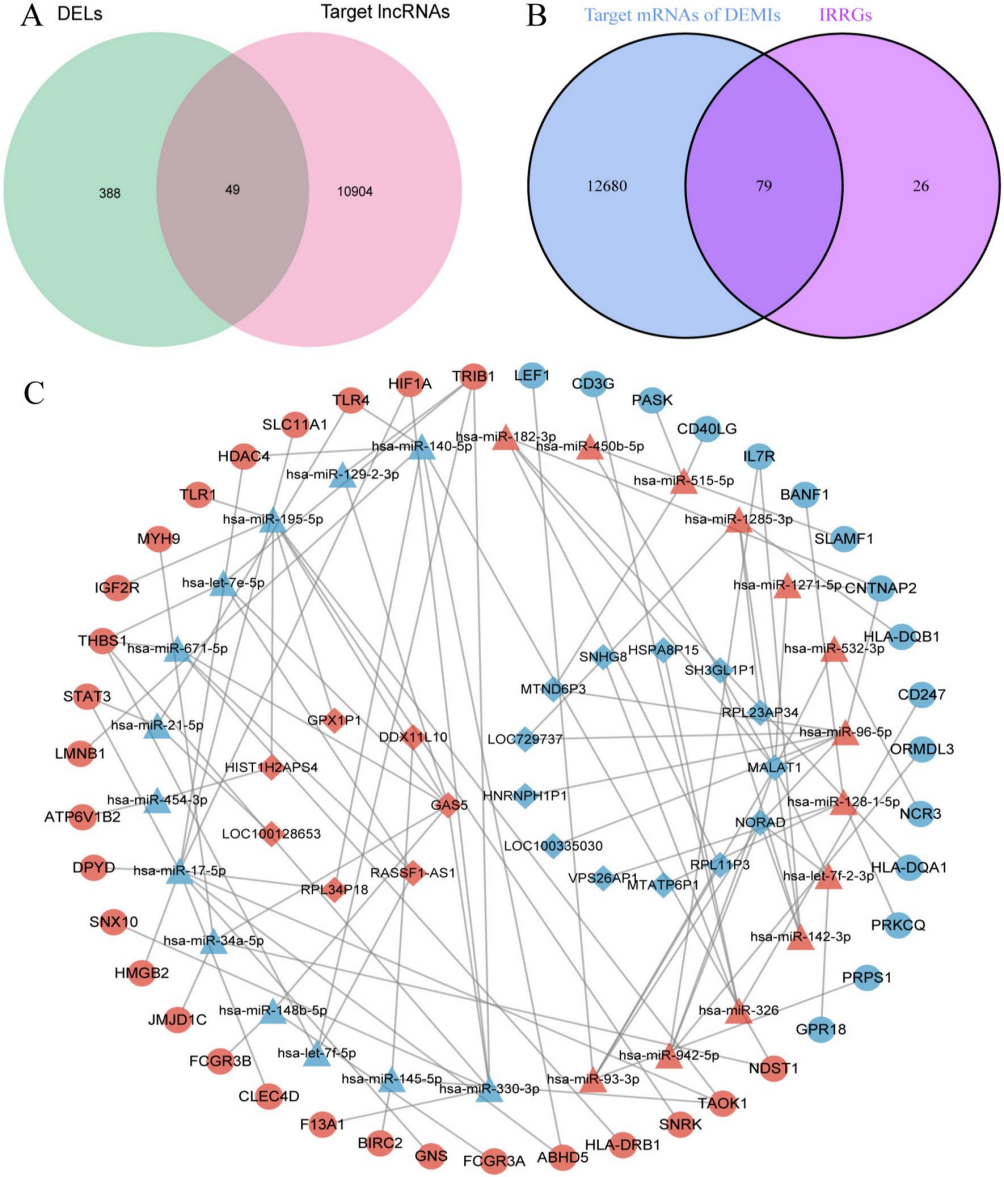
Construction of IRRCN. (**A**) Intersecting of DELs and target lncRNAs to obtain DEL-DEMI pairs. (**B**) Intersecting of DEMs and target mRNAs to obtain DEMI-DEM pairs. (**C**) IS-related lncRNA-miRNA-mRNA regulatory network, edges represent interactions between genes, diamonds represent lncRNAs, triangles represent miRNAs, and circles represent mRNAs. Up-regulation is represented by red, while down-regulation is represented by blue.

To explore the regulatory relationship between DELs, DEMIs, and DEGs, a ceRNA network was established. This led to the identification of 49 intersecting lncRNAs in the integration of DELs and target lncRNAs of DEMIs from the lncBase database (Fig. 2A). In addition, 79 intersecting genes were identified in the integration of IRRDEGs and target genes of DEMIs from miRDB and Targetscan databases (Fig. 2B). Subsequently, we constructed a lncRNA-miRNA-gene ceRNA network using the aforementioned relationship pairs. Overall, 106 edges and 89 nodes were identified in the network, among which 13 were down-regulated lncRNAs, 7 were up-regulated lncRNAs, 13 were down-regulated miRNAs, 13 were up-regulated miRNAs, 20 were down-regulated genes, and 23 were up-regulated genes (Fig. 2C).

### Functional enrichment analysis

**Fig. 3:**
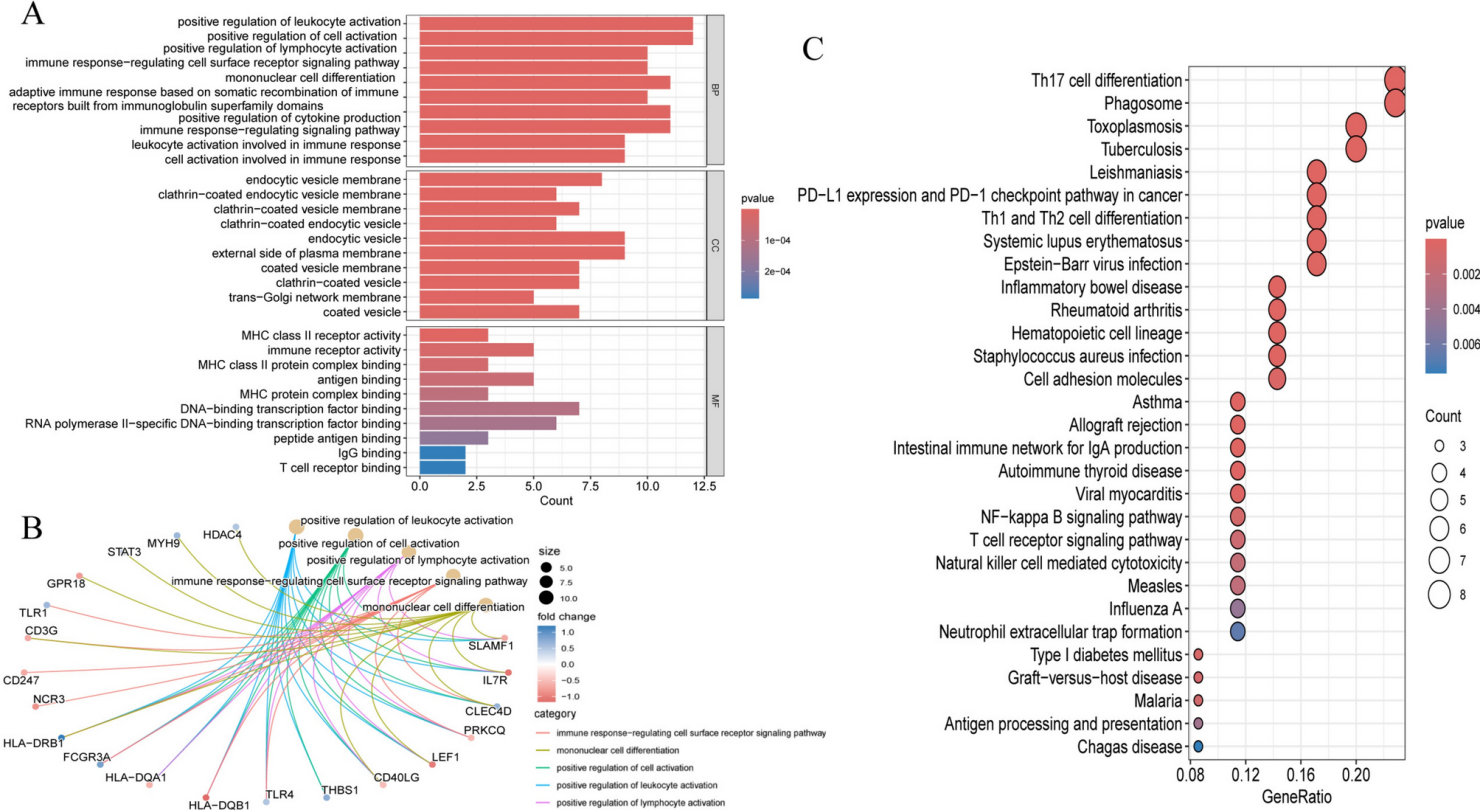
GO and KEGG analyses. (**A**) Top ten enriched terms for BP, CC, MF. (**B**) Network plot of enriched terms. (**C**) KEGG enrichment analysis.

Further GO and KEGG enrichment analyses of 43 genes in the network were conducted to predict their potential biological processes and pathways. A *p*-value < 0.05 was set to select significant processes and the top 10 items were displayed. In the biological process (BP) category, the genes were enriched in the positive regulation of leukocyte activation, positive regulation of cell activation, and positive regulation of lymphocyte activation. For the cellular component (CC), the genes were mainly enriched in endocytic vesicle membrane, clathrin−coated endocytic vesicle membrane, and clathrin−coated vesicle membrane. In the molecular function (MF) category, they were primarily enriched in MHC class II receptor activity, immune receptor activity, and MHC class II protein complex binding (Fig. 3 A,B). KEGG results further indicated that these genes were strongly enriched in the Th17 cell differentiation, Phagosome, and Th1 and Th2 cell differentiation (Fig. 3C).

### PPI analysis and identification of IRRHGs

**Fig. 4:**
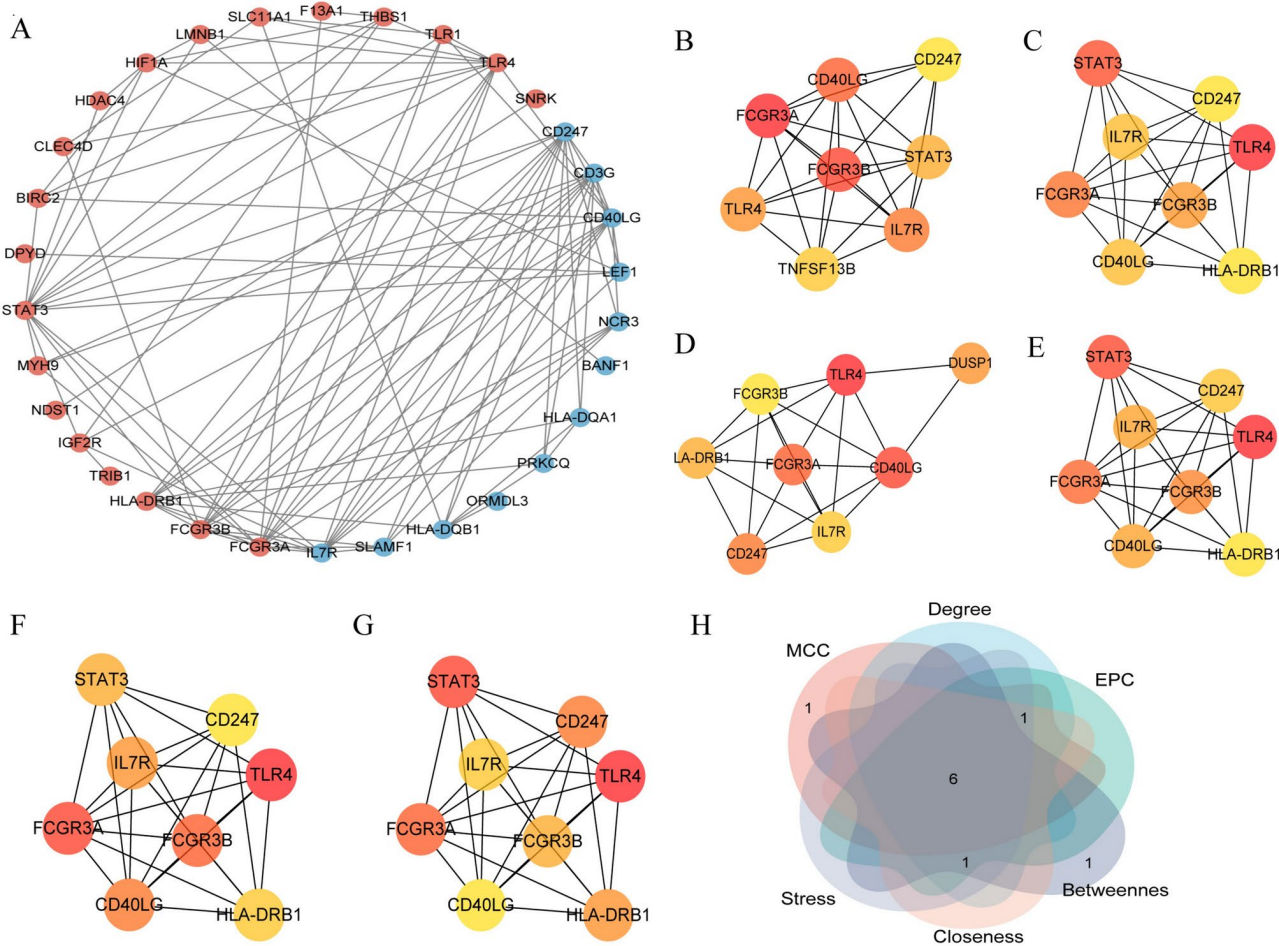
PPI network analysis and identification of IRRHG. (**A**) PPI network of mRNAs in the IRRCN. (**B**) Top 8 mRNAs arranged by MCC score. (**C**) Top 8 mRNAs arranged by degree score. (**D**) Top 8 mRNAs arranged by Betweenness score. (**E**) Top 8 mRNAs arranged by Closeness score. (**F**) Top 8 mRNAs arranged by EPC score. (**G**) Top 8 mRNAs arranged by Stress score. (**H**) Intersection of the 6 topological features.

Using the STRING database, we constructed a PPI network using 34 genes in the network. Following the removal of the disconnected nodes, 32 nodes and 101 edges were obtained (Fig. 4A). By intersecting 6 topological features, 6 genes were calculated as IRRHGs (Fig. 4B). These genes were found to be strongly associated with the inflammatory response: FCGR3A, FCGR3B, and CD247 are linked to natural killer cell-mediated cytotoxicity; IL7R is involved in the cytokine-cytokine receptor interaction; and TLR4 and STAT3 are associated with the HIF-1 signaling pathway. This suggests a high probability of their involvement in the inflammatory response.

### Extraction of hub-gene subnetworks and diagnostic performance of key biomarkers

**Fig. 5:**
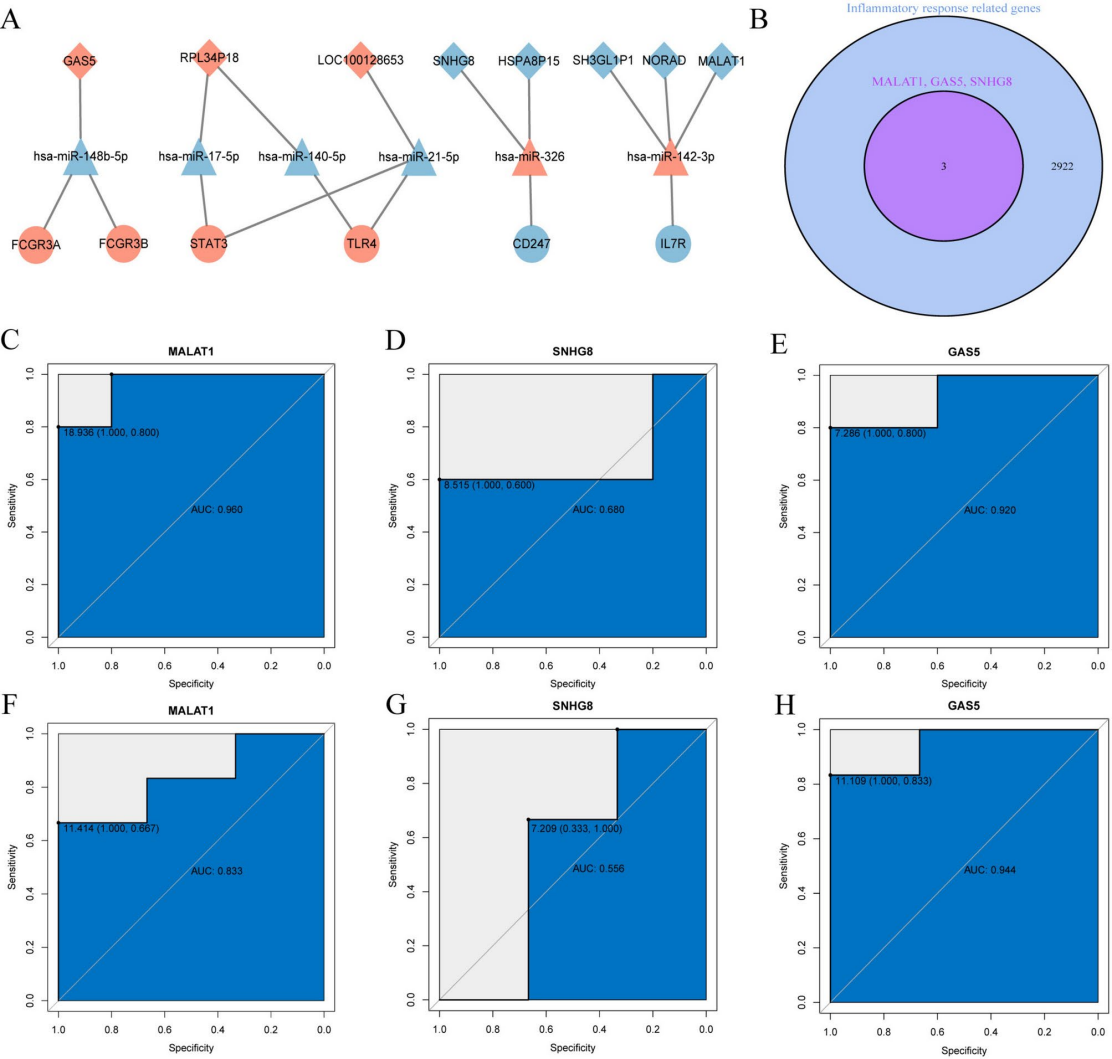
IRRHG subnetwork extraction and identification of key biomarkers. (**A**) IRRHG subnetwork. (**B**) MALAT1, GAS5, SNHG8 were found in the IRRGs (**C**) Diagnostic efficacy of MALAT1 in discovered dataset (GSE122709). (**D**) Diagnostic efficacy of SNHG8 in discovered dataset (GSE122709). (**E**) Diagnostic efficacy of GAS5 in discovered dataset (GSE122709). (**F**) Diagnostic efficacy of MALAT1 in validation dataset (GSE102541). (**G**) Diagnostic efficacy of SNHG8 in validation dataset (GSE102541). (**H**) Diagnostic efficacy of GAS5 in validation dataset (GSE102541).

To identify inflammation-related DELs, the above two IRRHGs as seed nodes and two lncRNA-miRNA-IRRHG subnetworks were obtained from the ceRNA network (Fig. 5A). Six down-regulated lncRNAs, 1 up-regulated lncRNA, 1 down-regulated miRNA, 3 up-regulated miRNAs, and 5 IRRHGs were included in the subnetwork. Analysis of the network revealed that lncRNAs (MALAT1, SNHG8, GAS5) were present in the IRRGs (Fig. 5B). In addition, we considered the aforementioned lncRNAs as potential inflammatory response-related diagnostic biomarkers in AIS, and explored their diagnostic performance using the ROC analysis on the discovered dataset (GSE122709) and validation set (GSE102510). Notably, the AUC value of the 3 lncRNAs was 0.960 (MALAT1, 95% CI 0.800–1.000), 0.680 (SNHG8, 95% CI 0.600–1.000), and 0.920 (GAS5, 95% CI 0.800–1.000) in the discovery dataset (Fig. 5C–E).In comparison, the AUC value of the 3 lncRNAs was 0.833 (MALAT1, 95% CI 0.667–1.000), 0.556 (SNHG8, 95% CI 0.333–1.000), and 0.944 (GAS5, 95% CI 0.833–1.000) in the validation set (Fig. 5F–H). Among them, MALAT1 and GAS5 were deemed to be potential candidates as diagnostic biomarkers.

### Differentially expressed lncRNAs in mice MCAO model

**Fig. 6:**
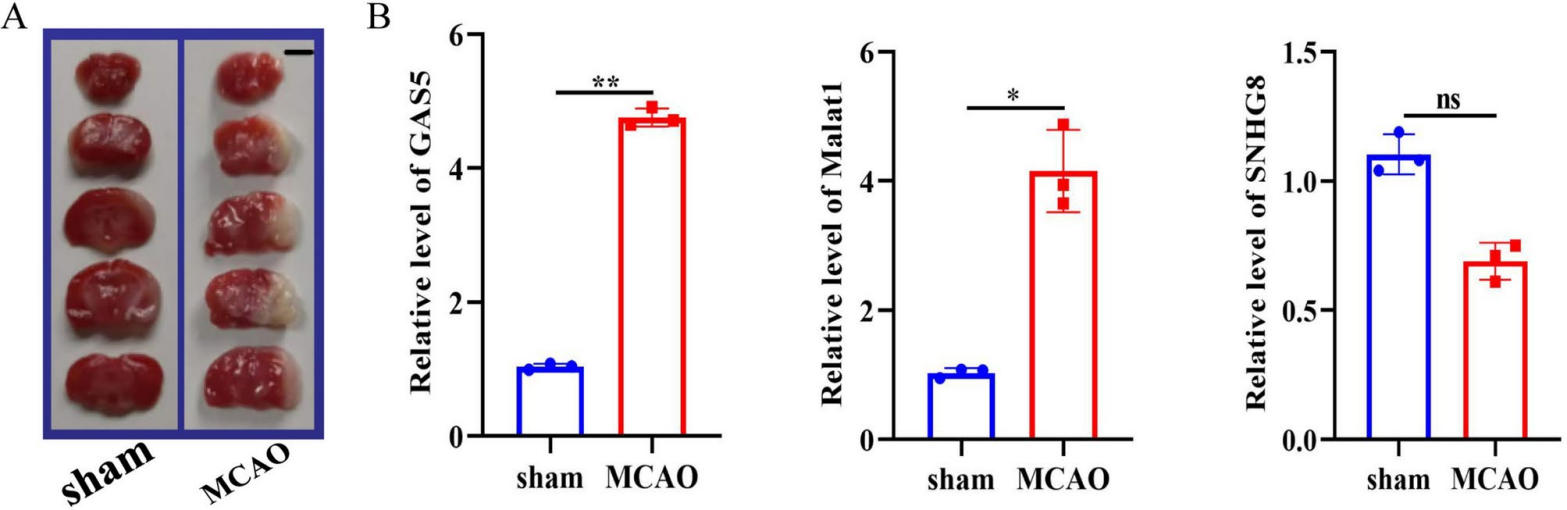
Validation of critical lncRNAs in the MCAO model. (**A**) TTC staining of mice MCAO model; (**B**) qRT-PCR verification of critical lncRNAs between sham and MCAO group. Mean ± SEM, n = 3, *p < 0.05, **p < 0.01, vs sham group.

Subsequently, we established mice models of MCAO (simulating AIS) to investigate the diagnostic potential of the lncRNAs in AIS. Results of the TTC staining assay for cerebral tissues demonstrated the success of the model (Fig. 6A). Further measurement of the expression levels of candidate lncRNAs by qRT-PCR in samples from the mice ischemic penumbra and the sham group revealed that GAS5 and MALAT1 were significantly up-regulated in the MCAO mode group than in the sham group. However, SNHG8 was not significantly different between the two groups (Fig. 6B).

### Validation of critical lncRNAs highly associated with inflammation in mice mcao model

**Fig. 7:**
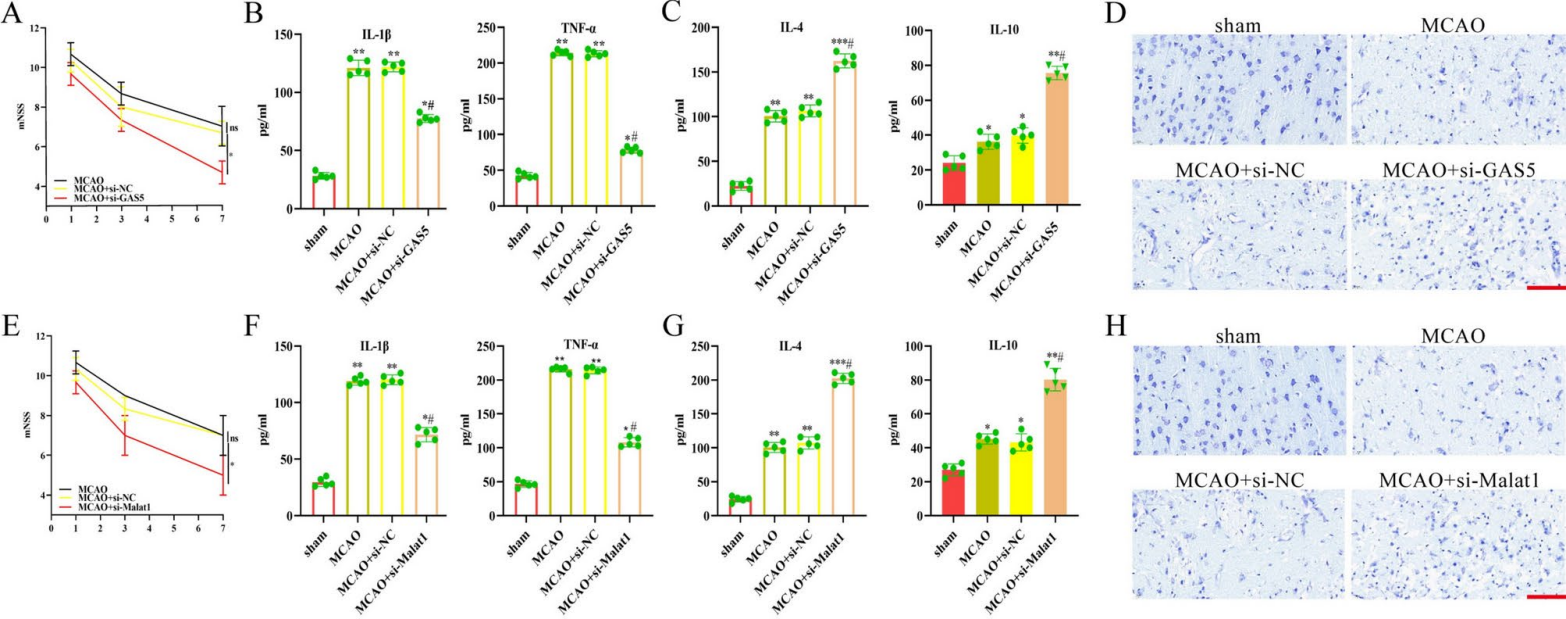
GAS5 and Malat1 knockdown dropped proinflammatory cytokines after MCAO. (**A**) Time course of mNSS with the knockdown of GAS5 in different groups. (**B**) The protein levels of IL-1βand TNF-αin the infarcted cortex were detected with ELISA. (**C**) The protein levels of IL-4 and IL-10 in the infarcted cortex were detected with ELISA. (**D**) Nissl staining of the ischemic cerebral cortex of sham, MCAO, MCAO + si-NC and MCAO + si-GAS5 groups. (**E**) Time course of mNSS with the knockdown of Malat1 in different groups. (**F**) The protein levels of IL-1βand TNF-αin the infarcted cortex were detected with ELISA. (**G**) The protein levels of IL-4 and IL-10 in the infarcted cortex were detected with ELISA. (**H**) Nissl staining of the ischemic cerebral cortex of sham, MCAO, MCAO + si-NC and MCAO + si-MALAT1 groups. The scale bar represents 50 μm. Data are presented as the mean ± SD (**A**–**C**, **E**–**G**), n = 5, *p < 0.05, **p < 0.01, ***p < 0.001, vs sham group. #p < 0.05, vs MCAO + si-NC group, Mann–Whitney test.

To further validate the role of GAS5 and MALAT1 through the mice MCAO model, lentivirus sh-GAS5 and lentivirus sh-MALAT1 were transfected to knock down GAS5 and MALAT1 expression in MCAO mice. The success of GAS5 knockdown was successful as shown in (Fig. S1A). Compared to the sh-NC group, the sh-GAS5 group showed significantly decreased GAS5 expression. Furthermore, the sh-GAS5 group exhibited less functional damage compared to the sh-NC group (Fig. 7A). Results of the ELISA indicated that GAS5 knockdown resulted in the suppression of MCAO-induced proinflammatory cytokine levels (IL-1β and TNF-α) (Fig. 7B) and enhanced the anti-inflammatory cytokines levels (IL-10 and IL-4) (Fig. 7C). The Nissl’s staining further showed that the MCAO + si-GAS5 group showed better neuronal survival and less damage relative to the MCAO group (Fig. 7D). Similarly, the successful knockdown of MALAT1 was confirmed through qRT-PCR (Fig. S1B). The sh-MALAT1 group had lower functional damage compared to the MCAO and sh-NC groups (Fig. 7E). It was also observed that MALAT1 knockdown reduced MCAO-induced proinflammatory cytokine levels (IL-1β and TNF-α) and upregulated anti-inflammatory cytokines levels (IL-10 and IL-4) (Fig. 7F,G). In the Nissl’s staining, the MCAO + si-MALAT1 group had better neuronal survival and less damage compared to the MCAO group (Fig. 7H). These findings suggested that GAS5 and MALAT1 knockdown reduces the inflammatory response to alleviate cerebral ischemic injury.

### Differentially expressed lncRNAs in the clinical samples

**Fig. 8:**
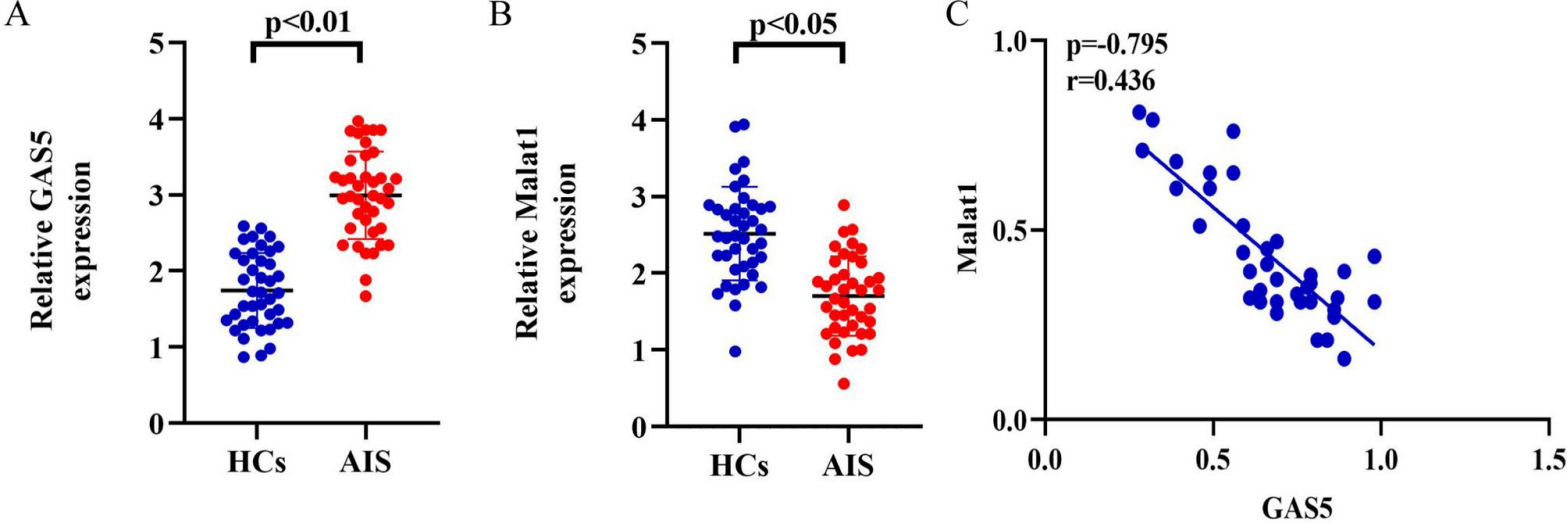
Relative levels of (**A**) GAS5 and (**B**) Malat1 via qRT-PCR in the clinical samples. (**C**) Correlation between GAS5 and Malat1 expression in AIS patients and HCs. n = 30, the data were all measurement data and manifested as mean ± SD, Kruskal–Wallis ANOVA test followed by Bonferroni correction pairwise comparisons.

To further validate the two lncRNAs, we measured the circulating levels of GAS5 and MALAT1 using clinical samples, which included 40 AIS patients and 40 HCs. The validation samples from both HCs and AIS patients were well-matched in terms of their clinical characteristics. The detailed baseline characteristics of the enrolled samples are shown in (Table 2). Compared with the HCs, the expression levels of circulating MALAT1 were significantly elevated in AIS patients, while the expression of circulating GAS5 was decreased (Fig. 8A,B). Furthermore, a negative correlation was observed between the expression levels of the above lncRNAs (Fig. 8C).

**Table 2:** Baseline characteristics of the validation.

Baseline characteristics	Validation sample		
	IS	HCs	*P*-value
Total, n	40	40	–
Age, mean (SD),years	62.5(12.7)	62.7(12.9)	0.234
Female, n (%)	18(46.4%)	19(53.6%)	0.050
Dyslipidemia	12(32.1%)	13(28.6%)	0.058
Coronary artery disease	7(14.3%)	8(17.9%)	0.082
Hypertension	23(64.3%)	21(57.1%)	0.202
Atrial fibrillation	6(14.3%)	4(10.7%)	0.457
Diabetes mellitus	9(21.4%)	8(25.0%)	0.075
Alcohol	8(17.9%)	10(28.6%)	0.287
Smoking history	9(25.0%)	10(28.6%)	0.069
Total cholesterol, mmol/L	4.46(1.25)	4.38(1.14)	0.683
HDL, mmol/L	1.20(0.42)	1.25(0.59)	0.172
LDL, mmol/L	2.77(0.71)	2.78(0.69)	0.459
Triglycerides, mmol/L	2.39(0.91)	4.48(0.93)	0.448
Serum glucose, mmol/L	5.51(2.49)	5.53(1.87)	0.153

### Diagnostic performance of GAS5 and MALAT1 for AIS

To dissect the diagnostic value of GAS5 and MALAT1 for AIS, univariate and multivariate logistic regression analyses were conducted using the aforementioned clinical samples. Results of the univariate analyses revealed independent correlation between AIS and the two lncRNAs. After adjustment for clinical factors (age and sex), biochemical indexes and vascular risk factors on admission, multivariate analysis revealed that the expression levels of GAS5 and MALAT1 were correlated with AIS. The odds ratios (ORs) and 95% CIs of the two lncRNAs for AIS are shown in (Table 3).

**Table 3:** Logistic regression analysis for predictive power of GAS5 and MALAT1.

Variable	Univariate analysis		Multivariate analysis	
	OR (95% CI)	P-value	OR (95% CI)	P-value
GAS5	2.353(2.278–4.593)	< 0.001	2.795(1.362–4.548)	< 0.001
MALAT1	3.531(1.268–4.329)	< 0.001	2.821(1.754–3.436)	< 0.001

**Fig. 9:**
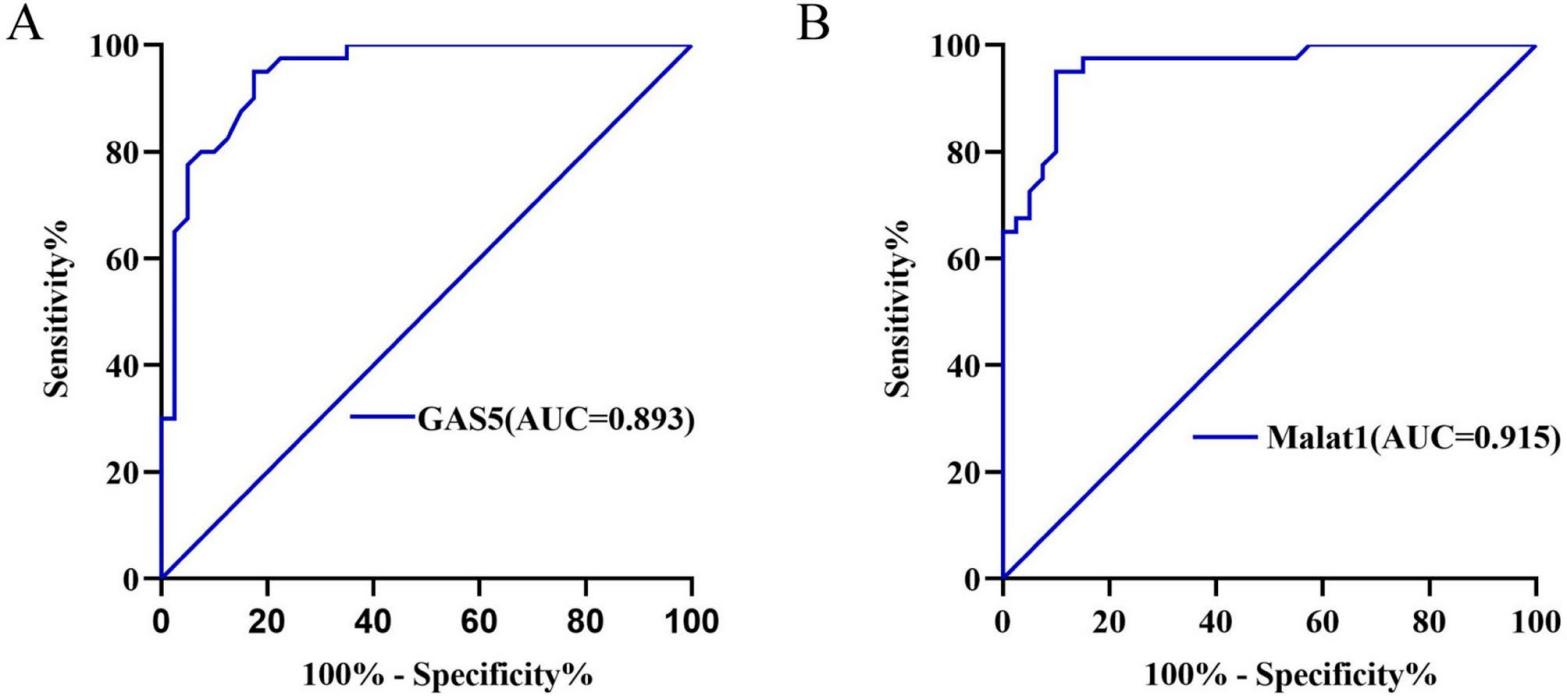
Diagnostic value of individual lncRNA. ROC curves were calculated with the baseline levels of lncRNAs to discriminate AIS patients from HCs. (**A**) GAS5, (**B**) Malat1.

In the ROC analyses using the same clinical samples, the AUCs of GAS5 and MALAT1 in distinguishing AIS from HCs were 0.893 (95% CI 0.826–0.898) and 0.915 (95% CI 0.828–0.931), respectively (Fig. 9 and Table 4).

**Table 4:** Diagnostic property of individual circRNAs.

Variables	AUC(95% CI)	P-value	Sensitivity	Specificity
GAS5	0.893 (0.826–0.898)	< 0.001	0.836	0.872
MALAT1	0.915 (0.828–0.931)	< 0.001	0.732	0.813

## Discussion

Acute ischemic stroke (AIS) is a cerebral disease characterized by inadequate blood supply to the brain due to formation of a thrombus or embolism. During its initial phase, inflammation and maladaptive immune responses contribute significantly to the pathologic process^25,26^. Therefore, it is imperative to explore the role of inflammation and immunity as biomarkers for identifying patients at high risk and establish innovative approaches for preventing and treating AIS. Recently, ncRNAs have received significant interest owing to their role in the pathogenesis and development of AIS. For instance, circRNA circOGDH has been shown to regulate the expression of COL4A4 by interacting with mir-5512, to promote neuronal activity, serving as a diagnostic biomarker of AIS^27^. Furthermore, the study showed that SNHG15, which regulates inflammation, can inhibit inflammation by enhancing TRAF2 ubiquitination, serving as a potential target for therapeutic interventions in AIS^28^. However, the potential of lncRNAs as diagnostic biomarkers for AIS remains largely unexplored. To address this gap and further investigate the role of lncRNAs in AIS, we constructed a lncRNA-mediated ceRNA network. This network was designed to identify inflammation-related lncRNAs associated with AIS and to evaluate their diagnostic utility in distinguishing AIS patients from HCs.

Currently, non-contrast head CT scans are the primary tools for the diagnosis of AIS at the time of admission. To date, several early CT image signatures for AIS patients have been documented^29^. However, the known imaging markers are not sufficiently sensitivity during the first stage of AIS. Moreover, although MRI is more effective than non-contrast head CT in identifying ischemic lesions in their early stages, it is expensive and not easily accessible. Furthermore, MRI necessitates longer scanning durations and is contraindicated for patients with metallic foreign objects or implanted devices^30^. Therefore, there is a need for significant research to identify novel diagnostic markers to accelerate rapid and accurate differentiation of AIS patients, enabling timely and effective treatment of AIS.

Studies have shown that ceRNA is a noncoding regulatory mechanism with two notably advantages. For instance, it regulates brain damage in the early stages of AIS^26^. Second, in the ceRNA network, a competitive interaction occurs between mRNAs and noncoding RNAs through their shared miRNA response elements (MREs). LncRNAs, by competing for the same miRNAs, can regulate mRNA expression. This mechanism underscores the fact that transcriptional regulation is more effective than translational regulation at the RNA expression level^31^. By modulating miRNAs, lncRNAs serve as biomarkers and participate in the regulation of gene expression^32^. Recent advances in the development of detection technologies have led to the identification of circulating lncRNAs involved in AIS inflammation^33^, making them promising candidates for diagnostic biomarkers.

In this study, we constructed an AIS-associated ceRNA network consisting of 26 DELs, 20 DEMIs, and 43 DEGs, based on the identification of 437 DELs, 215 DEMIs, and 527 DEGs from the GEO dataset. GO enrichment analysis revealed that most DEGs were strongly associated with inflammation-related BPs, such as the positive regulation of leukocyte, cell, and lymphocyte activation. Further, KEGG pathway analysis indicated significant enrichment in inflammation-related pathways, including Th17 cell differentiation, phagosome, and Th1/Th2 differentiation. Moreover, three lncRNAs strongly linked to inflammation in AIS were identified via the lncRNA-miRNA-IRRHG sub-network. The diagnostic value of the validation and discovery sets was assessed through ROC analysis. Moreover, the expression and inflammatory roles of MALAT1 and GAS5 were analyzed in MCAO mice models and clinical samples, confirming their potential as diagnostic biomarkers for circulating inflammation-related responses.

Two lncRNAs (MALAT1 and GAS5) in the DEL-DEMI-IRRHG subnetwork were identified, which may serve as inflammatory-related diagnostic biomarkers for AIS. These lncRNAs were associated with the MALAT1-hsa-mir-142-3p-IL7R and GAS-hsa-mir-148b-5p-FCGR3A/FCGR3B axes. Among them, MALAT1 is an evolutionarily conserved and stable lncRNA which is extensively characterized in multiple species^34^. A prior study showed that it was significantly upregulated in MCAO brain tissues and OGD/R-treated astrocytes, where it promoted inflammation and apoptosis^35,36^. However, in peripheral blood of AIS patients, it was downregulated and negatively correlated with the prognosis, indicating its complex regulatory mechanism^37^. In MCAO models, MALAT1 was upregulated in cerebral microvascular endothelial tissues, inhibiting inflammation, whereas in brain tissue, it stimulated inflammation via the signaling miR-145/AQP4 pathway^36,38^. These differences in the function and distribution underscore the need for further investigation into the regulatory mechanisms of MALAT1 in different tissues. GAS5 is a lncRNA that is highly expressed in growth-arrested cells and regulates cellular sensitivity to apoptosis and other growth-related signals^39^. Research data indicate that GAS5 can downregulate the IL-10 expression, contributing to its pro-inflammatory role^40^. Moreover, it is elevated in AIS patients, and positively related to the disease severity and inflammation^41^. In MCAO mice, GAS5 stimulates the release of pro-inflammatory factors via the miR-9/FOXO3 axis^42^ and activates inflammation and apoptosis through the miR-21b-5p/Smad1 axis^43^. Moreover, both lncRNAs can affect AIS-associated inflammatory responses. In this study, MALAT1 and GAS5 showed good performance in the diagnosis of AIS, making them ideal diagnostic biomarkers for adoption in clinical practice. Moreover, the two lncRNAs were significantly correlated with the occurrence of inflammatory responses in the mice MCAO model, which highlights their potential as robust indicators for assessing the severity and prognosis of AIS patients.

This study has several limitations that need to be discussed. Firstly, the sample size of each dataset used in this study was relatively small. Further, high-throughput sequencing experiments with large samples are advocated to validate the present findings. In addition, given that the datasets used in this study were from diverse test platforms, which may introduce bias, despite efforts to minimize it in the R package. Moreover, the clinical validation was based on a small cohort, and thus large multicenter studies are needed to obtain more comprehensive data. Therefore, future research is required to expand the current understanding of the role of the lncRNA-mediated ceRNA axis in the regulation of inflammatory response in AIS.

## Supplementary Information

Supplementary Information.

## Data Availability

The data that support the findings of this study are available from the corresponding author upon reasonable request.

## Abbreviations

IS: Ischemic stroke
AIS: Acute ischemic stroke
CT: Computed tomography
MRI: Magnetic resonance imaging
ceRNA: Competing endogenous RNAs
miRNA: MicroRNA
MREs: MiRNA response elements
ncRNAs: Noncoding RNAs
lncRNA: Long noncoding RNA
HCs: Health controls
GEO: Gene expression omnibus
ROCs: Receiver operating characteristic curves
MCAO: Middle cerebral artery occlusion
DELs: Differentially expressed lncRNAs
DEMIs: Differentially expressed miRNAs
DEGs: Differentially expressed genes
IRRDEGs: Inflammatory response-related differentially expressed genes
IRRCN: Inflammatory response-related ceRNA network
GO: Gene ontology
KEGG: Kyoto encyclopedia of genomes
PPI: Protein-protein interactions
IRRHGs: Inflammatory response-related hub genes
AUC: Area under the curve
CI: Confidence intervals
TTC: 2,3,5-Triphenyltetrazolium chloride
qRT-PCR: Quantitative real-time PCR
GAS5: Growth arrest specific 5
MALAT1: Metastasis associated lung adenocarcinoma transcript 1
mNSS: Modified neurological severity score
TNF-α: Tumor necrosis factor alpha
IL-1β: Interleukin 1 beta
IL-4: Interleukin 4
IL-10: Interleukin 10
BP: Biological process
CC: Cellular component
MF: Molecular function
ORs: Odds ratios
