# Supplementary material for: Identification and validation of the inflammatory response-related LncRNAs as diagnostic biomarkers for acute ischemic stroke
Source: Sci Rep. 2025 Apr 22;15:13818. doi: 10.1038/s41598-025-98101-0 (PMC12012103; doi:10.1038/s41598-025-98101-0)

**Table S1 PCR primers sequences**

|  | Forward sequence | Reverse sequence |
| --- | --- | --- |
| GAS5 | 5′-GCTTGAGGAGGAGTCTGA-3′ | 5′-GGTCTTCTATTCTAGCACATTG-3′ |
| MALAT1 | 5′-AGGCAGGTGGGAGATGAT-3′ | 5′-GGTCTGTGCTAGATCAAAAGGC-3′ |
| SNHG8 | 5'‐CACGTGGCGGTAAGAGCTCTGCTTCAG‐3’ | 5’‐TTTGGCTCAGGCACTTCCTTCGTGC‐3’ |
| β-actin | 5'-CGCGAGTACAACCTTCTTGC-3' | 5'-CGTCATCCATGGCGAACTGG-3' |

Abbreviations: GAS5, growth arrest specific 5; MALAT1, metastasis associated lung adenocarcinoma transcript 1; SNHG8, small nucleolar RNA host gene 8; β-actin as the internal control.

**Table S2 PCR primers sequences**

|  | Forward sequence | Reverse sequence |
| --- | --- | --- |
| GAS5 | 5'- TGTGGCTCTGGATAGCACCTTA -3' | 5'- GTCTAATGCCTGTGTGCCAATG -3' |
| MALAT1 | 5'- GAGGTGTTTGATGACCCGTTTA -3' | 5'- GACGGAGAACAACTCGCATCA -3' |
| GAPDH | 5'- GTCTCCTCTGACTTCAACAGCG -3' | 5'- ACCACCCTGTTGCTGTAGCCAA -3' |

Abbreviations: GAS5, growth arrest specific 5; MALAT1, metastasis associated lung adenocarcinoma transcript 1; GAPDH as the internal control.

**Figure S1**

1. The level of GAS5 was measured with qRT-PCR. (B) The level of MALAT1 was measured with qRT-PCR. Data are presented as the mean±SEM (A and B).


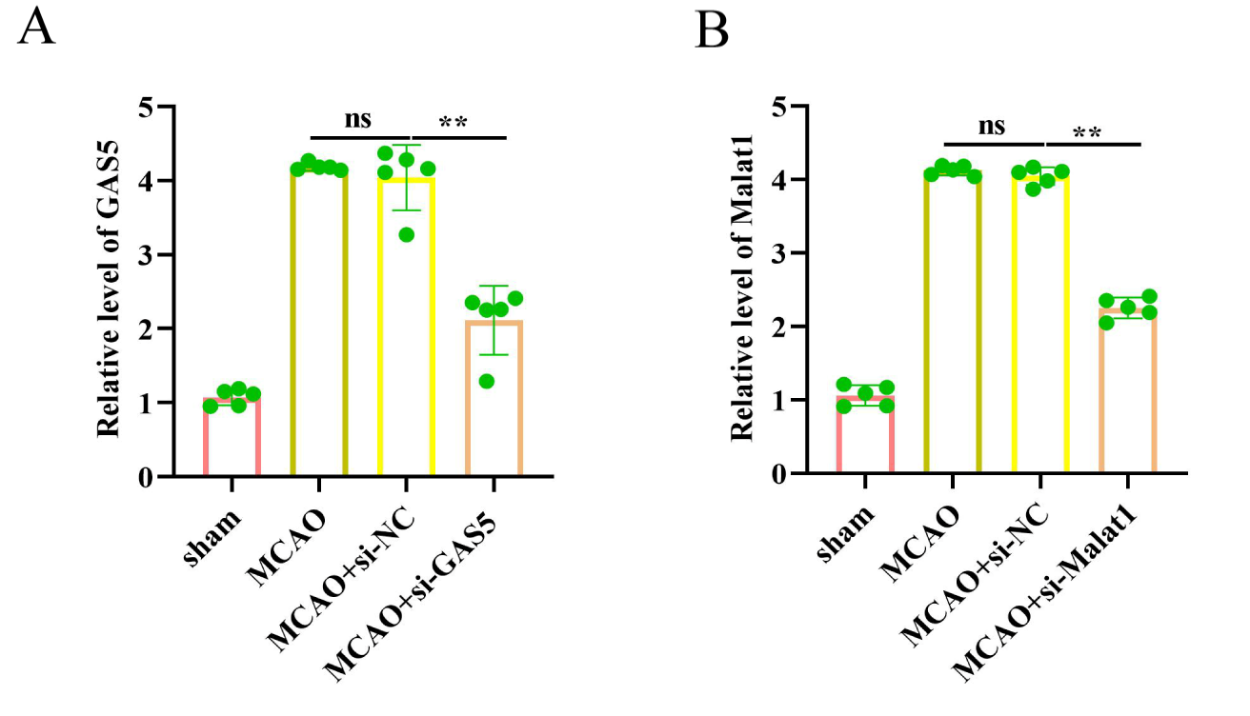

Supplement: Supplementary file 1 — Supplementary Information. [file 41598_2025_98101_MOESM1_ESM.docx]
